# Hospital Contracts and Prices

**Published:** 1910-06-11

**Authors:** 


### Special Departmental Article.

Contributions and questions on matters affecting this department of an institution are invited.

### HOSPITAL CONTRACTS AND PRICES.

### IX.—CLEANING ARTICLES, FUEL, &c., UNIFORMS, AND PRINTING.

#### Cleaning Articles.

The majority of the cleaning articles will, of course, be included in the grocery contract. Some hospitals make a practice of purchasing soap direct from the manufacturers, a three or six months' quantity being purchased at one time. Others are content to include all soaps in the grocery tender. Too cheap a quality is not economical for laundry purposes. Individual hospitals have preferences for different manufactures, and it is probable that a particular soap may suit the water of a locality better than that of another make. There are many good makers whose soap can be relied upon, and whether hospitals purchase direct or through a local firm, if a reliable brand be selected the institution cannot make a serious mistake.

#### Fuel and Water.

Fuel is one of the least satisfactory articles to purchase. There are so many different qualities, so many different collieries and seams, and such difference in the rate of combustion that in the case of steam coal the furnace in which it is to be burnt, the height of the chimney, the amount of draught, etc., have all to be considered in selecting the coal. A coal which burns cleanly under certain conditions will clinker and be unsatisfactory under other conditions, and hospital authorities should undertake tests until they find a coal which suits the furnace in which it has to be burnt and which proves economical. June is usually considered a good month in which to arrange an annual contract. Prices must vary according to the distance of the institution from the coalfields, but for a large contract it is usually found that the competition is keen and that prices are cut as finely as possible.

#### Gas and Electricity.

Gas has perforce to be obtained from the local authority or company, and it is seldom that special terms can be arranged.

Electricity can either be obtained from the local authority or company, or a plant for its production can be installed, and the possibility of the latter is a factor which may reasonably be considered by the local authority or company in fixing the rate to be charged for a supply to a large charity. There is frequently a desire to help charities in these hard times which is productive of favourable consideration when points such as the one under reference are being dealt with by electricity committees of corporations, and hospital committees who consider that too high a rate per unit is being charged for the supply to the institution might, with a distinct prospect of advantage, approach the local authority on the subject.

#### Uniforms.

Nurses' and servants' uniforms are dealt with in a variety of ways. Sometimes the materials are purchased in bulk and issued to scale to the nurses and servants concerned. Sometimes the dress materials only are dealt with in this way, and the caps and aprons issued ready-made from the institution's sewing-room. Sometimes nurses' uniforms are issued in material, and servants' dresses are entirely made for them, the understanding being that the uniform remains the property of the hospital, and is returned when the maid leaves.

The cost of materials varies according to the ideas of the different hospital authorities. Suitable prints can be purchased at about 5d. per yard, galateas or sateen shirtings at about 7d., an excellent calico for aprons at about 9d., nainsook for caps at 7d. An annual purchase of materials required for the year insures close prices from the competing firms.

The cost of porters' uniforms varies according to the style adopted. A livery with coloured facings may cost £5 or £6 per suit, whereas a braided patrol jacket suit can be purchased for from £2 15s. to £3 5s., and the porters will still look smart and soldierly; if the uniform is of serge, and buttoned instead of braided, it will cost less and prove serviceable.

#### Theatre Uniforms.

Most of the theatre uniform, such as aprons, overalls, leggings, veils, etc., for surgeons, caps and gowns for nurses, etc., have to be made in the hospital workroom, surgeons having their own special patterns which have to be followed. The material should be purchased at the same time as the hospital linen supply and included in the same contract. Rubber gloves, included under this sub-head, are difficult to purchase at the present time, so are the surgeons' theatre boots, the great increase in the cost of rubber having inflated prices of manufactured goods considerably. It is to be hoped that the formation of the numerous rubber companies and the establishment of fresh plantations will result in a few years in more favourable terms being obtainable.

#### Printing and Stationery.

The last subject to which we have to refer in this series of articles is "Printing and Stationery." Every hospital has its own series of forms and books, and it is difficult to suggest prices for these goods. An annual contract, selected firms being invited to tender to definite copies and samples, is a very good method of purchasing; and a five years' contract, with a definite schedule of forms, power of revision from time to time, without increase of price, being reserved, is another and less troublesome method, and one which can be heartily commended from an economical point of view.

